# Remissions and Relapses in Insular Sclerosis

**Published:** 1904-09-10

**Authors:** 


### REMISSIONS AND RELAPSES IN INSULAR SCLEROSIS.

In a clinical lecture Dr. Buzzard[1] expresses the opinion that sufficient prominence has not been given to the more or less frequent remissions and recurrences of symptoms which may mark the course of insular sclerosis. Amongst other cases he relates one which included such symptoms as transitory numbness, diplopia, and short-lived but recurrent facial paralysis among the earlier events, these completely or in a large measure disappearing before definite disturbance of gait was observed some two years later. Similar remissions may be noted in connection with disturbances of the functions of the bladder, and even in paretic developments in the limbs. It is the failure to appreciate such facts as possible incidents in the course of insular sclerosis that has led to many such cases being diagnosed and treated as examples of hysteria. In one of Dr. Buzzard's cases a parallel experience was encountered in reference to the function of one of the optic nerves. The patient, a woman of 22 years, while in excellent health observed dimness of vision in her left eye and the visual acuity was found to be only $\frac{6}{60}$, the eye being emmetropic and the fundus normal. At the same time the left hand and arm were numbed and there was some disturbance of gait. In the following year the vision was normal, but there was nystagmus with numbness in both hands and below the left knee, and an extensor response (Babinski's sign) was obtained on each side on stimulation of the sole. Later the patient was able to hunt and was reported to be "quite well." Dr. Buzzard suggests that the visual disturbance was due to a transitory retro-ocular neuritis occurring in the course of insular sclerosis, and whilst it is possible that no new development may occur the possibility of a relapse cannot be excluded. In another instance double vision and squinting were present at 13 years of age and persisted for three months, when they completely disappeared. Three years later the sight of the left eye failed, and it was feared the patient would become blind, but in the end nearly full visual acuity returned. Afterwards there was again diplopia on three separate occasions, each attack extending over several weeks. Yet a few years later the patient was so well that she was able to walk for 10 hours a day. Subsequently, however, and about 18 years after the early symptoms, she had a characteristic staggering gait and the usual exaggeration of the tendon phenomena.

An important question arises from these clinical facts—namely, whether they throw any light on the mode of the causation of the disease. The most likely view regarding the cause of insular sclerosis is that of an infective agent present in the blood and capable of producing at any point in the cerebro-spinal axis an inflammatory process in the interstitial tissue. The suggestion that there is an underlying congenital abnormality is negatived by the gross character of the anatomical changes and by the extreme rarity of the occurrence of the disease in several members of the same family. It seems reasonable to hold, too, that the fact of variations in the degree of acuteness in insular sclerosis favours the theory of an infective agent rather than that of congenital defect. In examples of the latter, such as Friedreich's ataxia, the movement is chronic and practically uninterrupted. In insular sclerosis, on the other hand, as illustrated above, there may be marked remissions, and, whilst many cases extend over 10 or even 20 years, there are others in which a fatal issue follows the initial symptoms after the lapse of only a few months.

